# A Metabolic Trade-Off Modulates Policing of Social Cheaters in Populations of *Pseudomonas aeruginosa*

**DOI:** 10.3389/fmicb.2018.00337

**Published:** 2018-02-27

**Authors:** Huicong Yan, Meizhen Wang, Feng Sun, Ajai A. Dandekar, Dongsheng Shen, Na Li

**Affiliations:** ^1^School of Environmental Science and Engineering, Zhejiang Gongshang University, Hangzhou, China; ^2^Zhejiang Provincial Key Laboratory of Solid Waste Treatment and Recycling, Hangzhou, China; ^3^Department of Microbiology, University of Washington, Seattle, WA, United States; ^4^Department of Medicine, University of Washington, Seattle, WA, United States

**Keywords:** quorum sensing, LasR, RhlR, sociomicrobiology, hydrogen cyanide

## Abstract

*Pseudomonas aeruginosa* uses quorum sensing (QS) to regulate the production of public goods such as the secreted protease elastase. *P. aeruginosa* requires the LasI–LasR QS circuit to induce elastase and enable growth on casein as the sole carbon and energy source. The LasI–LasR system also induces a second QS circuit, the RhlI–RhlR system. During growth on casein, LasR-mutant social cheaters emerge, and this can lead to a population collapse. In a minimal medium containing ammonium sulfate as a nitrogen source, populations do not collapse, and cheaters and cooperators reach a stable equilibrium; however, without ammonium sulfate, cheaters overtake the cooperators and populations collapse. We show that ammonium sulfate enhances the activity of the RhlI–RhlR system in casein medium and this leads to increased production of cyanide, which serves to control levels of cheaters. This enhancement of cyanide production occurs because of a trade-off in the metabolism of glycine: exogenous ammonium ion inhibits the transformation of glycine to 5,10-methylenetetrahydrofolate through a reduction in the expression of the glycine cleavage genes *gcvP1 and gcvP2*, thereby increasing the availability of glycine as a substrate for RhlR-regulated hydrogen cyanide synthesis. Thus, environmental ammonia enhances cyanide production and stabilizes QS in populations of *P. aeruginosa*.

## Introduction

Bacterial quorum sensing (QS) regulates group behaviors. *Pseudomonas aeruginosa* uses QS to regulate the production of a variety of extracellular products, which can be shared “public goods” in population of cells (Diggle et al., 2007; Wilder et al., 2011; Stevens et al., 2012). There are two acyl-homoserine lactone (AHL) QS circuits in *P. aeruginosa*, the LasI–LasR circuit and the RhlI–RhlR circuit. LasI is an enzyme that catalyzes synthesis of the diffusible QS signal *N*-3-oxo-dodecanoyl-homoserine lactone (3OC12-HSL). At sufficient concentrations, 3OC12-HSL can bind to the transcription factor LasR, which in turn activates a constellation of genes including *rhlR* and *rhlI*. The RhlI–RhlR circuit consists of butanoyl-homoserine lactone (C4-HSL), produced by RhlI, and the C4-HSL-dependent RhlR (Schuster et al., 2003; Gilbert et al., 2009), which activates a set of genes that partially overlap with LasR-dependent genes.

Some quorum-regulated secreted products have been established as public goods that are shared among all cells in a population (Diggle et al., 2007; Pollitt et al., 2014). Growth of *P. aeruginosa* with casein as the sole source of carbon and energy requires production of the QS-regulated protease elastase (Diggle et al., 2007), which digests casein into peptides and amino acids that can be taken up and used by *P. aeruginosa* cells regardless of whether they produce elastase themselves. When *P. aeruginosa* is grown continuously on casein, LasR-mutant cheaters, which produce neither elastase nor other quorum-regulated factors, emerge (Sandoz et al., 2007; Dandekar et al., 2012).

Uncontrolled cheating can result in a tragedy of the commons wherein there are insufficient numbers of cooperators to maintain growth on casein (Diggle et al., 2007; Dandekar et al., 2012; Wang et al., 2015). Mechanisms for cheater control have been described and include kin-selection (Diggle et al., 2007; Allen et al., 2013), prudent regulation of costly metabolism (Xavier et al., 2011), metabolic constraints (Dandekar et al., 2012), and metabolic policing (Wang et al., 2015).

When *P. aeruginosa* is grown on casein as the sole carbon and energy source and ammonium sulfate as additional nitrogen source, cheaters emerge and eventually equilibrate with cooperators at 25–40% of the total population (Sandoz et al., 2007; Dandekar et al., 2012). However, when casein is the sole nitrogen as well as carbon source, cheater frequency rises to 80% or more, ultimately leading to a tragedy of the commons (Dandekar et al., 2012). Establishment of the cooperator–cheater equilibrium in media containing ammonium sulfate depends on RhlR-dependent hydrogen cyanide (HCN) production by the cooperators (Wang et al., 2015), as deletion of *hcnC*, a component of the HCN synthase, or *rhlR* results in cheaters outcompeting cooperators. The cooperators presumably regulate both cyanide production and a detoxification mechanism through *rhl* QS (Wang et al., 2015). The production of cyanide by cooperators in these conditions serves as a policing strategy to enforce cooperation (West et al., 2007). There is a cost (in both growth rate and final cell density) to the wildtype (w.t.) in generation of HCN (Wang et al., 2015). However, the production of HCN imparts a disproportionate fitness impact on cheaters (i.e., they are more susceptible to cyanide intoxication).

We are interested in the costs and benefits of cooperation in bacteria and asked if there is an interaction between exogenous ammonia and production of cyanide in casein medium. One possible link is through glycine degradation: *P. aeruginosa* converts glycine to cyanide (Castric, 1977; Devi et al., 2013); alternatively, glycine can be degraded to ammonia and CO_2_ by glycine dehydrogenase, which is encoded by *gcvP1* and *gcvP2* (Toone et al., 2002; Kai et al., 2009), in a process involving tetrahydrofolate and NADH (Goro et al., 2008). *gcvP2*, but not *gcvP1*, is also LasR-regulated (Schuster et al., 2003). We reasoned that ammonia in the environment might alter the outcome of glycine metabolism to favor cyanide production, and therefore improve the ability of *P. aeruginosa* to restrain cheaters in media containing ammonium sulfate. We show that ammonium sulfate enhances cyanide production by *P. aeruginosa*. These studies help us understand how *P. aeruginosa* senses its environment to stabilize QS and give insight into the development and stabilization of cooperative behavior in bacteria.

## Materials and Methods

### Bacterial Strains and Media Used

We used the following *P. aeruginosa* strains in our experiments: PAO1-UW- (Stover et al., 2000) and PAO1-UW-derived deletion mutants of *rhlR*, *lasR*, and *hcnC* (Wang et al., 2015). Bacteria were grown in either LB media buffered with 50 mM 3-(*N*-Morpholino) propane-sulfonic acid (MOPS), pH 7.0 (LB-MOPS) (Sambrook et al., 1989) or in photosynthetic medium (PM) (Kim and Harwood, 1991) supplemented with 1% sodium caseinate (Dandekar et al., 2012). The base PM-casein medium contains 1% ammonium sulfate, which we reduced or increased as described in individual experiments. Unless otherwise specified, cultures were grown in 4 ml of PM-casein medium in 16-mm test tubes with shaking at 37°C.

### Evolution Experiments

Inoculation for experiments in PM-casein medium was from overnight LB-MOPS cultures (Sambrook et al., 1989). The initial optical density was 0.025 at 600 nm. Subsequently, we transferred 200 μl at 24 h intervals to fresh casein broth for days 1–3; subsequent transfers were 50 μl daily (Wang et al., 2015). Cheater abundance was determined by plating on skim milk agar, as previously described (Dandekar et al., 2012); we enumerated at least 100 colonies for each condition at each timepoint. For competition experiments, overnight cultures of PAO1 and PAO1Δ*lasR* were mixed at ratios of 99:1 or 9:1, and used to inoculate PM-casein medium as described above. Cheater frequency was determined daily at the time of passage.

### Dialysis Membrane Competition Experiments

Strain PAO1Δ*lasR* was sealed in a dialysis bag in 5 ml of PM-casein medium at an initial density of 0.025. Strain PAO1 was used to inoculate 45 ml of PM-casein medium (starting OD_600_ of 0.025) in a 250 ml flask. The dialysis bag containing PAO1Δ*lasR* was placed into the PAO1 culture flask which was then incubated at 37°C with shaking (250 rpm). We verified that PAO1Δ*lasR* did not leak from the dialysis bag by phenotype screening (Dandekar et al., 2012).

### Reporter Assays

We used *lasI-gfp* (pBS351) and *rhlA-gfp* promoter fusion constructs (pJF01) (Feltner et al., 2016) in pPROBE-GT (Gm^r^) (Miller et al., 2000) to measure LasR and RhlR activity, respectively. Cells from overnight cultures were electrotransformed with either plasmid after preparation with 300 mM sucrose (Choi et al., 2006). Transformants were selected on LB agar containing 100 mg ml^-1^ gentamicin. GFP activity was determined after 24 h of incubation by adding 200 μl culture to black 96-well plates and measuring fluorescence in a SpectraMax^®^ i3 Plate Reader (Molecular Devices, Sunnyvale, CA, United States) with 488 nm excitation and 525 nm detection wavelengths.

### RT-qPCR

Cells from either a mixed culture or the outside of the dialysis bag were pelleted by centrifugation at 16,430 × *g* for 3 min at 4°C to collect cells. RNA isolation and cDNA synthesis were performed as described elsewhere (Fey et al., 2004). Primers for *lasR*, *rhlR*, *gcvP1*, *gcvP2*, *hcnC*, and *proC* are shown in **Table 1**. qPCR reactions were carried out in 96-well plates with a CFX96 real-time PCR detection system (Bio-Rad, Hercules, CA, United States) using SYBR premix Ex Taq II Kit (TaKaRa, Japan). After 40 cycles, a melting curve analysis was performed (60–95°C) to verify the specificity of amplicons. Each amplification was repeated three times. The specificity of the amplicons was confirmed by the presence of a single peak. The relative level of expression (*Q*) for a given gene was calculated based on the formula *Q* = 2^-ΔCt^. Each Ct value represents the average of three replicates, where ΔCt represents the target gene Ct minus the Ct of the housekeeping gene *proC* (Kim et al., 2017).

**Table 1:** Primers used in this study.

Target gene	Primer name	Sequence (5′–3′)	Amplicon (bp)	*T*_m_ (°C)	Function
*lasR*	q*lasR*-F	5′-cagaagatggcgagcgaccttg-3′	102	59.9	QS regulator
	q*lasR*-R	5′-cgggtagttgccgacgatgaag-3′		59.7	
*rhlR*	q*rhlR*-F	5′-gcgaccagcagaacatctccag-3′	122	59.6	QS regulator
	q*rhlR*-R	5′-cgggttggacatcagcatcgg-3′		59.6	
*hcnC*	q*hcnC*-F	5′-gccagtacgccgagcacatc-3′	145	59.6	Hydrogen cyanide synthase
	q*hcnC*-R	5′-acctggtggtcgcagaggaatt-3′		59.9	
*gcvP1*	q*gcvP1*-F	5′-cgctgctgaatttccagcaa-3′	142	55.5	Glycine cleavage system protein P1
	q*gcvP1*-R	5′-aagaaccggttgctcctggc-3′		58.6	
*gcvP2*	q*gcvP2*-F	5′-cgctgctgaacttccagacc-3′	142	57	Glycine cleavage system protein P2
	q*gcvp2*-R	5′-aatgcctggctggtgcggtt-3′		61	
*proC*	q*proC*-F	5′-cgtcgtggtcctgtcggtca-3′	100	59.9	House-keeping gene
	q*proC*-R	5′-ggcggcgatggagacgatca-3′		60.4	

### Reactive Oxygen Species Measurement

Strains PAO1, PAO1Δ*rhlR*, or PAO1Δ*hcnC* were mixed with strain PAO1Δ*lasR* at a 9:1 ratio in casein broth with or without ammonium sulfate. After 24 h, the supernatant fluid was collected after centrifugation at 16,430 × *g* for 3 min and 4°C. The supernatant was added to an equal volume of a PAO1Δ*lasR* culture in logarithmic phase (OD_600_ = 1.0). Cell density was measured every 2 h.

To measure ROS, 2′,7′-dichlorofluorescin diacetate (DCFH-DA) was added to the PAO1Δ*lasR* culture with different supernatants at a 10 mM final concentration. After incubating at 37°C for 90 min in dark, cells were washed in PBS twice and resuspended in 1 ml PBS. Reaction of DCFH-DA with ROS generates a fluorescent, oxidized derivative, DCF (F). DCF (F) levels were measured in a SpectraMax^®^ i3 Plate Reader with 488 nm excitation wavelength and 525 nm detection wavelength. We calculated the relative ROS dividing the DCF (F)/OD_600_ of experimental samples by that of fresh PM-casein without supernatant.

### Ammonia Accumulation

PAO1 was mixed with PAO1Δ*lasR* at a 9:1 ratio in casein broth with or without ammonium sulfate. We centrifuged 2 ml of culture fluid at 16,430 × *g* for 3 min. The abundance of ammonia in the supernatant was measured according to Standard Methods American Public Health Association (Federation and American Public Health Association [APHA], 2005). Briefly, we added 1 ml of the sample to a 50 ml cuvette and diluted to 25 ml with distilled water. Then we added 5 ml of phenol solution (10 g l^-1^ phenol and 0.1 g l^-1^ sodium nitroprusside) and 5 ml of sodium hypochlorite alkaline solution (10 g l^-1^ NaOH, 7.06 g l^-1^ KH_2_PO4, 31.8 g l^-1^ Na_3_PO_4_, 10 ml l^-1^ NaClO), for 1 h. Next, we added 1 ml of masking agent (200 g l^-1^ potassium sodium tartrate, 50 g l^-1^ EDTA disodium salt, and 2 g l^-1^ NaOH) and an additional 24 ml H_2_O. The absorbance was then measured at 625 nm using a spectrophotometer.

### Hydrogen Cyanide Measurements

We modified the method of Gallagher and Manoil (2001) to measure HCN. Briefly, cultures were grown for 24 h at 37°C with shaking in 16 mm tubes with a rubber stopper. Two needles were inserted into the rubber stopper. Nitrogen gas entered through one needle, the other needle served as an outflow for gas collection. The collected gas was passed through 5.0 ml of 4 M NaOH in a cyanide collection tube. Cyanide levels were assessed by comparison with KCN standards. Cyanide production is expressed as the amount (μM) of HCN per cooperator. All experiments were performed in triplicate.

## Results

### Addition of Ammonium Sulfate Favors Cooperators in Competition with Cheaters

When w.t. *P. aeruginosa* PAO1 is grown in PM with casein as the sole carbon source (see section “Materials and Methods”), LasR-mutant social cheaters emerge and come to an equilibrium with the w.t. (Sandoz et al., 2007; Dandekar et al., 2012; Wang et al., 2015). However, when casein also serves as the sole nitrogen source, LasR mutants overrun the w.t. and cause a population collapse (Dandekar et al., 2012). This phenomenon has been attributed to the heightened cost of using QS to obtain carbon, energy, and nitrogen (Dandekar et al., 2012), and we reasoned that added ammonium sulfate might interact with QS-regulated gene products in a way that determined the fates of the populations.

**FIGURE 1:**
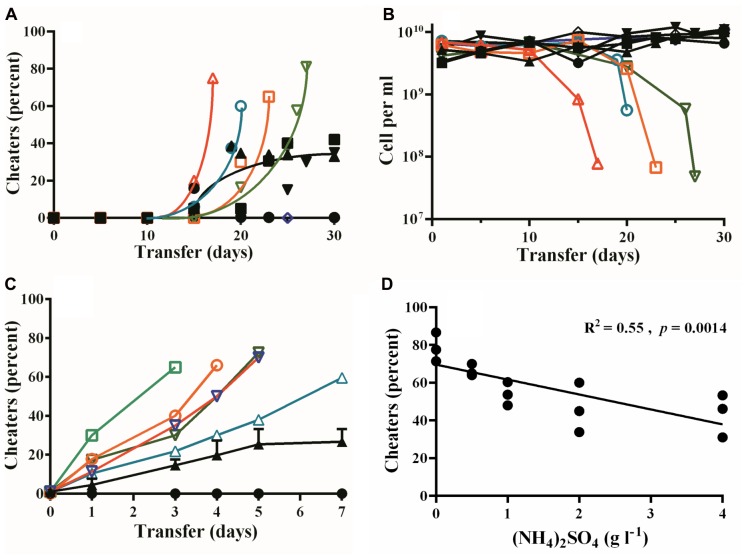
Emergence of LasR-mutant cheaters and their fitness in PM-casein with or without ammonium sulfate added. **(A)** Percent of protease-negative cheaters in PM-casein media over time. In four of five individual experiments without added ammonium sulfate, cheaters emerged and increased (hollow symbols). In PM-casein with ammonium sulfate (black symbols), cheaters reached a frequency of 27 ± 7%. In one experiment with and one experiment without ammonium sulfate, cheaters did not emerge. **(B)** Cell densities in casein experiments. In the cases where the culture could not be propagated, the final cell densities fell by 90–99% (hollow symbols). Experiments in PM-casein with ammonium sulfate (black symbols) are shown for comparison. **(C)** LasR-mutant frequency after 7 days of passage. LasR-mutant cheater frequency in PM-casein without ammonium sulfate was rapid in all five replicates from an initial frequency of 1% (hollow symbols). An equilibrium was always established (black triangles) in media with ammonium sulfate added. When LasR mutants were not present at the beginning of the experiment, they did not emerge in 7 days PM-casein with ammonium sulfate (black circles). **(D)** Outcomes of w.t. – LasR-mutant competitions with increasing ammonium sulfate concentrations. w.t. PAO1 and LasR-mutant cheaters were mixed at a 1:1 ratio and the frequency of each was enumerated after 24 h. Experiments were performed in triplicate. For the data in panel **D**, a linear regression analysis was performed.

Consistent with prior publications, when we grew PAO1 in PM-casein media with or without ammonium sulfate, LasR-mutant cheaters emerged by 15 days in four out of five experiments (**Figures 1A,B**). In PM-casein with ammonium sulfate, cheaters and cooperators reached a stable equilibrium as previously reported (Sandoz et al., 2007; Dandekar et al., 2012; Wang et al., 2015). In PM-casein without ammonium sulfate, however, the LasR-mutant frequency exceeded 60%, after which populations did not grow upon transfer.

To further examine the influence of ammonium sulfate on competition between w.t. and LasR mutants, we performed a 7-day competition between w.t. and LasR mutants. The inocula consisted of 99% w.t. and 1% LasR mutants. Consistent with prior reports (Sandoz et al., 2007; Dandekar et al., 2012), w.t. and LasR mutants reached a stable equilibrium of about 20% cheaters in PM-casein with ammonium sulfate. Without added ammonium sulfate, the LasR-mutant frequency in all five experiments increased rapidly and four of five populations collapsed (**Figure 1C**). This experiment is consistent with the results in **Figure 1A** and our previous report (Dandekar et al., 2012) that the presence of ammonium sulfate significantly reduced the fitness of LasR mutants in competition with the w.t.

### The Relative Fitness of LasR Mutants Is Dependent on the Concentration of Ammonium Sulfate

We next assessed the impact of ammonium sulfate on the cheater–cooperator equilibrium by performing 24 h competitions between the w.t. and LasR mutants. We started these competitions at initial ratios of 1:1 with variable amounts of added ammonium sulfate. After 24 h in ammonium sulfate-free PM-casein the frequency of cheaters rose to 79 ± 8%. At the maximum concentration of ammonium sulfate (4 g l^-1^), the frequency of cheaters decreased (albeit slightly) to 44 ± 11%. As the concentration of ammonium sulfate rose, the frequency of cheaters decreased (*R*^2^ = 0.55, *p* = 0.0014, **Figure 1D**).

### Ammonium Sulfate Enhances w.t. RhlR Activity in the Presence of LasR Mutant

Our observation that ammonium sulfate favored the w.t. (cooperators) over LasR mutants (cheaters) suggested that ammonium sulfate might influence the LasIR system, the RhlIR system, or both. As discussed above, both systems have a role in cheater–cooperator dynamics: the LasIR system regulates the public good elastase (Passador et al., 1993; Park et al., 2014), and RhlR regulates production of cyanide and perhaps other factors as a policing mechanism (Smalley et al., 2015; Wang et al., 2015).

**FIGURE 2:**
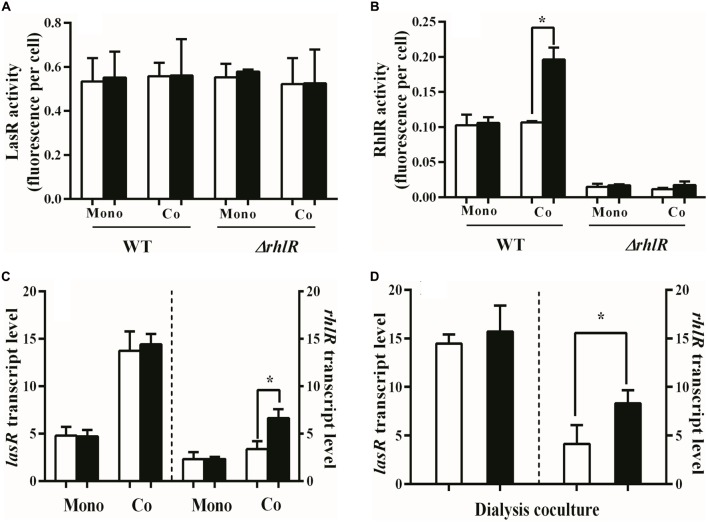
LasR and RhlR activities in w.t. *P. aeruginosa*. **(A)** GFP transcription in w.t. containing the *lasI–gfp* reporter plasmid pBS351. GFP is a proxy for LasR activity. Added ammonium sulfate does not alter the LasR activity of cooperators in w.t or a RhlR mutant, either in the presence or absence of cheaters. **(B)** GFP transcription in w.t. using the *rhlA–gfp* reporter plasmid pJF01. Addition of ammonium sulfate significantly increased the RhlR activity of cooperators in presence of cheaters. A RhlR mutant is shown as a reference. **(C)** Transcription of *lasR* and *rhlR* measured by qRT-PCR. Added ammonium sulfate significantly increased the transcript levels of *rhlR* but not *lasR* in the presence of cheaters. **(D)** Added ammonium sulfate significantly increased the expression of *rhlR* in the presence of LasR-mutant cheaters. The w.t. and LasR mutants were separated by a dialysis membrane. “Mono”: monoculture of w.t. or the RhlR mutant. “Co”: co-culture with 9:1 mixture of w.t and LasR mutants or a mixture of RhlR mutants and LasR mutants. We used *proC* as a reference (housekeeping) gene. White bars indicate PM-casein without ammonium sulfate and black bars indicate PM-casein with ammonium sulfate. Experiments were performed in triplicate. Data reported as the mean ± SD. ^∗^ indicates *p* < 0.05 by paired Student’s *t*-tests.

To test the hypothesis that ammonium sulfate enhances QS in the w.t., we used *gfp* fusion plasmids to monitor transcription from the *lasI* and *rhlA* promoters, as a proxy for LasR and RhlR activity (Feltner et al., 2016). Regulation of *lasI* and *rhlA*, unlike many QS-regulated genes, is specific to LasR and RhlR, respectively. Ammonium sulfate did not affect *lasI* or *rhlA* transcription in the w.t. when LasR mutants were not present (**Figures 2A,B**). Similar results were obtained with a RhlR-null mutant (**Figure 2A**). We reasoned that the w.t. might only increase QS in the presence of LasR mutants, as the presence of these cheaters increases the relative production of QS-regulated products by individual cooperators (Dandekar et al., 2012). Therefore, examined LasR and RhlR activity of the w.t. in the presence of 10% LasR mutants. We found that RhlR activity of the w.t. increased nearly twofold in the presence of LasR-mutant cheaters, but only in PM-casein with added ammonium sulfate (**Figure 2B**). The presence of LasR-mutant cheaters did not influence *lasI* transcription in the w.t.

The reporter experiments suggested that exogenous ammonium sulfate promotes RhlR, but not LasR, activity, only in presence of LasR-mutant cheaters. To test this idea further, we employed RT-qPCR to measure *lasR* and *rhlR* transcript levels in PM-casein with or without added ammonium sulfate, with and without LasR mutants. The presence of LasR mutants resulted in increased *lasR* transcription in the w.t., consistent with the previous observation of increased public-goods production on a per-cooperator basis (Dandekar et al., 2012); however, *lasR* transcript levels were unaffected by the presence of ammonium sulfate in PM-casein (**Figure 2C**). The expression of *rhlR* was induced by the presence of LasR-mutant cheaters, but only in the presence of added ammonium sulfate (**Figure 2C**). Finally, to exclude the possibility, however unlikely, that the w.t. can physically detect LasR mutants and activate policing in the presence of ammonium sulfate, we isolated the w.t. and LasR mutants using a dialysis membrane. We found the same dependence on ammonium sulfate (**Figure 2D**). Together, these experiments suggested that the presence or absence of exogenous ammonium sulfate was a cue for activation of the policing mechanism.

### Ammonium Sulfate Promotes Cyanide Production by Cooperators to Police Cheaters

**FIGURE 3:**
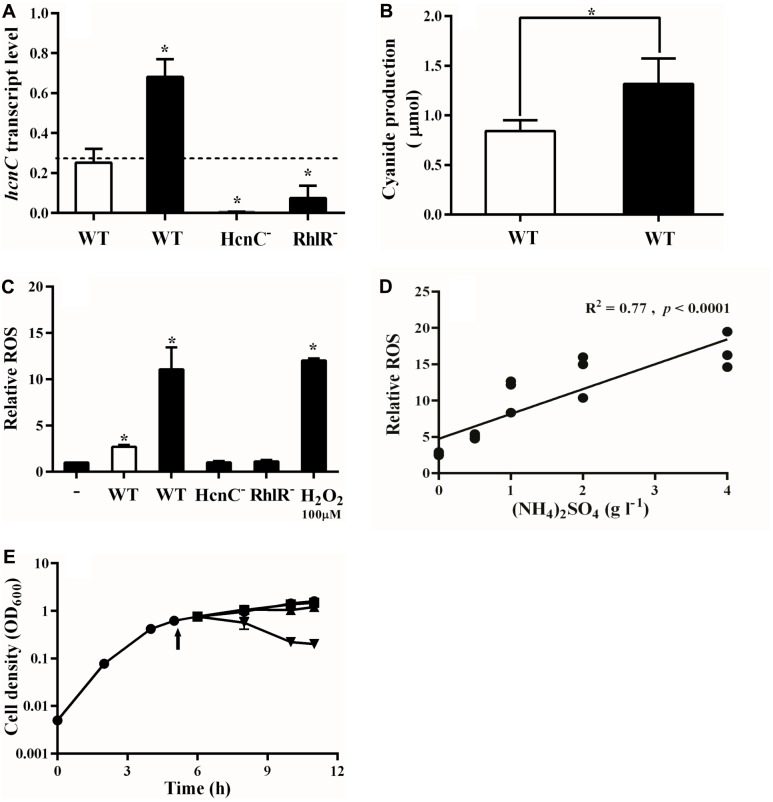
Ammonia promotes cyanide and ROS production by cooperators. **(A)** *hcnC* transcription in w.t. increases in presence of LasR-mutant cheaters in PM-casein with ammonium. *hcnC* expression from either a RhlR or HcnC deletion mutant in co-culture with the LasR mutant is shown as controls. *proC* was used as reference gene. In panels **A–C**, white bars indicate media without ammonium sulfate and black bars indicate that ammonium sulfate was added. **(B)** Cyanide production by cooperators in PM-casein with or without ammonium sulfate. **(C)** ROS production from LasR mutants in LB-MOPS, combined with supernatants of strains grown in PM-casein media. “-” indicates no cells. **(D)** ROS production by the w.t. increases as the concentration of ammonium sulfate is increased. **(E)** Growth inhibition of the LasR mutant by supernatant from w.t.:LasR-mutant co-culture from PM-casein with ammonia media (inverted triangle). The supernatant from a w.t.:LasR-mutant co-culture in PM-casein media (triangle) did not inhibit the growth of LasR mutants; neither did supernatant from a HcnC-mutant: LasR-mutant co-culture (square). The arrow indicates the time of supernatant addition. Experiments were performed in triplicate. ^∗^ indicates *p* < 0.05 by paired Student’s *t*-tests (panels **A–C** and **E**). A linear regression analysis was performed for the data presented in panel **D**.

In *P. aeruginosa*, cooperators produce cyanide to police social cheaters, which results in a population equilibrium between cooperators and cheaters in the conditions of our experiments (Wang et al., 2015). Based on the results above, we reasoned that ammonium sulfate might alter the amount of RhlR-dependent cyanide produced. To test this possibility, we measured expression of the HCN synthase gene *hcnC* in mixed populations of cooperators and cheaters in PM-casein, with or without ammonium sulfate and found that added ammonium sulfate increased w.t. *hcnC* expression by a factor of about 2 (**Figure 3A**). As expected, *hcnC* transcription was minimal in the RhlR mutant and undetectable in an HcnC deletion mutant (**Figure 3A**). We also measured cyanide levels in both conditions and found increased levels of PM-casein with ammonium sulfate (**Figure 3B**).

Cyanide inhibits cytochrome *c* oxidase in susceptible cells (Bernier et al., 2016), resulting in the production of reactive oxygen species (ROS) (Gallagher and Manoil, 2001; Hazan et al., 2016). We next asked if the cyanide-generated ROS inhibited the growth of cheaters (Garcia-Contreras et al., 2015). To do so, we analyzed effects of the supernatant from co-cultures of the w.t. and LasR mutant on the growth of LasR mutants. The ROS generated under these conditions paralleled the *hcnC* expression data (**Figure 3C**). The ROS generated by treatment of LasR mutants with supernatant from co-cultures in PM-casein with ammonium sulfate was nearly equal to that induced by addition of 100 μM of H_2_O_2_ in LB. Similar to *hcnC* expression, supernatant from co-cultures in PM-casein without ammonium sulfate only induced one-third of the ROS as those from where ammonium sulfate was added (**Figure 3C**). Is production of ROS by supernatant related to the concentration of ammonium sulfate in the medium? As the concentration of ammonium sulfate rose, so did ROS production, with a positive correlation (*R*^2^ = 0.77, *p* < 0.0001, **Figure 3D**).

As a final test of this hypothesis, we asked if co-culture supernatants could inhibit the growth of LasR-mutant cheaters. LasR-null mutants could not grow well in the supernatant from a co-culture grown in PM-casein with ammonium sulfate (**Figure 3E**), but the supernatant from a co-culture without ammonium sulfate did not inhibit growth of the LasR mutants. LasR-mutant cheaters also grew well in the supernatant from co-culture in which the cooperator was an HcnC mutant, in PM-casein with ammonium sulfate. From these data, we concluded that exogenous ammonium sulfate promotes cyanide production and enhances policing in a concentration-dependent manner. As such, populations of *P. aeruginosa* in media containing exogenous ammonium sulfate are more stable than those in PM-casein without ammonium sulfate.

### Ammonium Sulfate Increases the Transformation of Glycine to Hydrogen Cyanide

In *P. aeruginosa*, the main pathway to production of HCN is by using HCN synthase to catabolize glycine (Castric, 1977; Lenney and Gilchrist, 2011; Nandi et al., 2017). Glycine alternately can be converted to ammonia and 5,10-methylenetetrahydrofolate (5,10-methylene-THF) by the glycine cleavage system (Campbell, 1955; Toone et al., 2002). In *P. aeruginosa*, the glycine dehydrogenase is encoded by *gcvP1* and *gcvP2* (Kai et al., 2009; Zaara et al., 2016). The favored path of glycine catabolism might be determined through the abundance of substrates (van Niel et al., 2003; Neerincx et al., 2015). We hypothesized that the presence of ammonium sulfate in PM-casein medium inhibited the glycine cleavage pathway, thereby increasing HCN synthesis.

**FIGURE 4:**
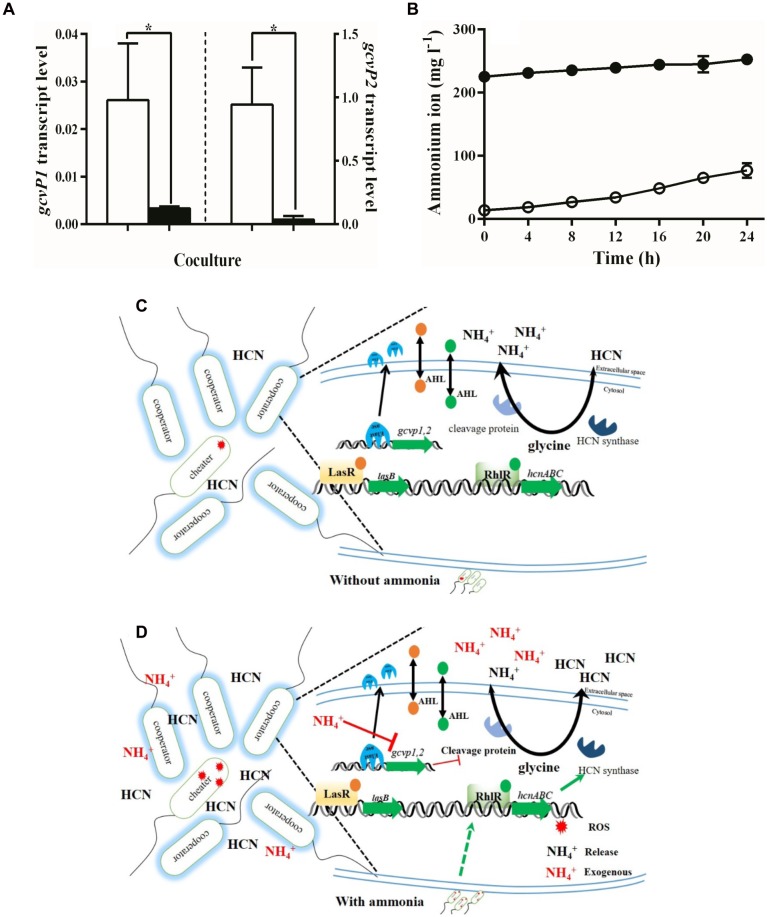
Exogenous ammonia inhibits the transformation from glycine to 5,10-methylene-THF. **(A)** Expression of *gcvP1* and *gcvP2* by the w.t. in a co-culture with 10% LasR mutants. Hollow bars indicate PM-casein without ammonium sulfate and black bars indicate PM-casein with ammonium sulfate. *proC* was used as a housekeeping gene. **(B)** PAO1 generates ammonia faster in the absence of ammonium sulfate (hollow circles) than in its presence (black circles). **(C)** Model of the flux between exogenous ammonia and cyanide production in *P. aeruginosa*. In panel **C**, red stars indicate ROS. The red NH_4_^+^ indicates exogenous ammonia in media, and black text indicates released ammonia by metabolism. Experiments were performed in triplicate. ^∗^ indicates *p* < 0.05 by paired Student’s *t*-test (panel **A**) or one-way ANOVA (panel **B**).

To test this possibility, we measured *gcvP1* and *gcvP2* transcription in the presence or absence of ammonium sulfate. We found that added ammonium sulfate decreased *gcvP1* transcription by w.t. PAO1 in the presence of 10% cheaters. *gcvP1* transcription in PM-casein with ammonium sulfate was only ∼12% of that in PM-casein without ammonium sulfate (**Figure 4A**). A similar reduction was seen in *gcvP2* expression (**Figure 4A**). Under both conditions, *gcvP2* expression was higher than *gcvP1*, consistent with evidence that *gcvP2* is LasR-regulated (Schuster et al., 2003). However, the reduction in expression of *gcvP2* in the absence of exogenous ammonium sulfate mitigates the QS-enhanced transcription of this gene. Combined with the *hcnC* expression and the cyanide production and relative ROS data above (**Figures 3A–C**), these results suggested that exogenous ammonium sulfate inhibits the glycine cleavage pathway, thereby favoring HCN synthesis.

An implication of this hypothesis is that more ammonia would be generated (from glycine) in PM-casein without ammonium sulfate. Therefore, we measured ammonium ion generation with and without added ammonium sulfate. In casein media with ammonium sulfate, little additional ammonia accumulated. However, in the absence of ammonium sulfate, the concentration of ammonium ion increased significantly (*p* = 0.0085, **Figure 4B**).

## Discussion

Quorum sensing, a cell–cell communication system, allows groups of bacteria to regulate a variety of biological processes important for adaptation and survival (Waters and Bassler, 2005; Xavier et al., 2011; Viducic et al., 2017) in response to cell density. Some of these processes are cooperative and therefore potentially vulnerable to social cheating (Joelsson et al., 2007; Wilder et al., 2011). Several mechanisms have been described that maintain cooperation in bacterial populations by which either invasion or growth of cheaters was restricted (Xavier et al., 2011; Dandekar et al., 2012; Darch et al., 2012; Pollitt et al., 2014; Wang et al., 2015; Allen et al., 2016).

When *P. aeruginosa* PAO1 is grown on casein as the sole carbon and energy source, LasR-mutant social cheaters emerge in the population. In our previous work, we showed that the presence of ammonium sulfate in casein broth leads to a stable frequency of cooperators and cheaters, while cheaters are uncontrolled in the absence of ammonium sulfate, leading to a tragedy of the commons (Dandekar et al., 2012). We used this system to study how environmental conditions might affect the stability of QS. We showed that the presence of ammonium sulfate results in upregulation of HCN synthesis, with the effect of policing social cheaters and stabilizing QS.

In our experiments, cheaters and cooperators co-existed stably in PM-casein with ammonium sulfate added but not in ammonium sulfate-free media (**Figures 1A–C**). The frequency of cheaters at the equilibrium was inversely correlated with the concentration of ammonium sulfate (**Figure 1D**). These finding extend our prior observation (Dandekar et al., 2012) that the absence of ammonium sulfate in the medium increases the per-cell cost of QS in *P. aeruginosa* to show that there is a biological effect of the ammonium sulfate in the growth medium.

RhlR activity among cooperators was significantly higher in PM-casein with ammonium sulfate than without (**Figures 2B–D**). Similarly, RhlR-dependent HCN production was significantly increased when ammonium sulfate was present in the media (**Figures 3A,B**). ROS generated as a consequence of HCN production also significantly increased (**Figures 3C,D**). Together, these finding suggested enhanced policing (Wang et al., 2015) by *P. aeruginosa* cooperators in the presence of ammonium sulfate.

The enhancement of cyanide production by ammonium sulfate appeared to involve a biochemical tradeoff. In *P. aeruginosa*, glycine is the main substrate for formation of cyanide (Castric, 1977); however, glycine cleavage results in ammonium ion production (Goro et al., 2008). We reasoned that the presence of exogenous ammonium sulfate reduces the need for glycine transfer to ammonia. In keeping with this hypothesis, the expression of *gcvP1* and *gcvP2*, genes whose products are involved in glycine cleavage, was inhibited in the presence of ammonium sulfate (**Figures 4A,B**). Conversely, HCN synthase gene expression was enhanced (**Figures 3A,B**). Thus, ammonium sulfate, through its modulation of the glycine cleavage pathway (**Figure 4C**), enhances policing in cooperating populations of *P. aeruginosa*.

There are other environmental conditions that have been reported to enhance HCN synthesis. For example, microaerophilic conditions and iron excess can have a stimulatory effect on cyanogenesis in *P. aeruginosa* (Blumer and Haas, 2000; Pessi and Haas, 2001). As HCN is an effector of policing in cooperating populations, these environmental conditions might also enhance the ability of populations to police themselves of cheaters.

Our experiments do not uncover the selective forces that gave rise to this mechanism of cheater control in the first place. It is notable that the mechanism does not require detection of cheaters *per se* but rather the abundance of ammonium ion in the growth medium. Although the presence of ammonium sulfate in our experiments affects the production of HCN by *P. aeruginosa* (**Figures 3**, **4**), it is most likely that this phenomenon simply reflects metabolic needs of the bacterium. However, it is possible that regulation of the HCN synthase by the QS regulator RhlR had the effect of selecting against LasR mutants in the population, providing stability to QS. Several studies have shown that cooperative behaviors (including QS) are stabilized by pleiotropy (Foster et al., 2004; Dandekar et al., 2012; Mitri and Foster, 2016); our results provide further support for this idea.

Our observation of ammonia-dependent elaboration of a QS-regulated product echoes the idea of “metabolic prudence” (Xavier et al., 2011). Part of the idea of metabolic prudence is that production of public goods should be deferred until the resources necessary for their production are abundant in the environment, thus minimizing the benefit of exploitation by cheaters. The production of rhamnolipid in *P. aeruginosa* occurs in this kind of prudent manner (Xavier et al., 2011). HCN production only when ammonia (the alternate fate of glycine) is abundant is similarly “prudent” for cooperators. However, in this case, the reduction in production of QS-regulated products, while possibly beneficial for cooperators in the short term, is ultimately deleterious for the population, and the production of HCN is lowest in the conditions where it is most needed (**Figure 3**). Although the cooperators may gain a short-term metabolic advantage when deprived of an exogenous nitrogen source, the population as a whole is prone to destabilization, ultimately resulting in the loss of cooperation. Further investigation into this mechanism might reveal methods to induce policing within or promote destabilization of a bacterial population, with ramifications for synthetic biological and clinical applications.

## Author Contributions

HY, MW, AD, and DS designed the research. HY and FS performed the research. HY, MW, AD, and NL analyzed the data. HY, MW, and AD wrote the paper.

## Conflict of Interest Statement

The authors declare that the research was conducted in the absence of any commercial or financial relationships that could be construed as a potential conflict of interest.

## References

[B1] Allen B. Nowak M. A. Wilson E. O. (2013). Limitations of inclusive fitness. *Proc. Natl. Acad. Sci. U.S.A.* 110 20135–20139. 10.1073/pnas.1317588110 24277847 PMC3864293

[B2] Allen R. C. Mcnally L. Popat R. Brown S. P. (2016). Quorum sensing protects bacterial co-operation from exploitation by cheats. *ISME J.* 10 1706–1716. 10.1038/ismej.2015.232 26744811 PMC4918439

[B3] Bernier S. P. Workentine M. L. Li X. Magarvey N. A. O’Toole G. A. Surette M. G. (2016). Cyanide toxicity to *Burkholderia cenocepacia* is modulated by polymicrobial communities and environmental factors. *Front. Microbiol.* 7:725. 10.3389/fmicb.2016.00725 27242743 PMC4870242

[B4] Blumer C. Haas D. (2000). Iron regulation of the *hcnABC* genes encoding hydrogen cyanide synthase depends on the anaerobic regulator ANR rather than on the global activator GacA in *Pseudomonas fluorescens* CHA0. *Microbiology* 146 2417–2424. 10.1099/00221287-146-10-2417 11021918

[B5] Campbell L. L. (1955). The oxidative degradation of glycine by a *Pseudomonas*. *J. Biol. Chem.* 217 669–674. 13271428

[B6] Castric P. A. (1977). Glycine metabolism by *Pseudomonas aeruginosa*: hydrogen cyanide biosynthesis. *J. Bacteriol.* 130 826–831. 233722 10.1128/jb.130.2.826-831.1977 PMC235287

[B7] Choi K. H. Kumar A. Schweizer H. P. (2006). A 10-min method for preparation of highly electrocompetent *Pseudomonas aeruginosa* cells: application for DNA fragment transfer between chromosomes and plasmid transformation. *J. Microbiol. Methods* 64 391–397. 10.1016/j.mimet.2005.06.001 15987659

[B8] Dandekar A. A. Chugani S. Greenberg E. P. (2012). Bacterial quorum sensing and metabolic incentives to cooperate. *Science* 338 264–266. 10.1126/science.1227289 23066081 PMC3587168

[B9] Darch S. E. West S. A. Winzer K. Diggle S. P. (2012). Density-dependent fitness benefits in quorum-sensing bacterial populations. *Proc. Natl. Acad. Sci. U.S.A.* 109 8259–8263. 10.1073/pnas.1118131109 22566647 PMC3361460

[B10] Devi K. K. Deepika S. Bhaduri A. Kothamasi D. (2013). Polymorphism in *hcnAB* gene in *Pseudomonas* species reveals ecologically distinct hydrogen cyanide-producing populations. *Geomicrobiol. J.* 30 131–139. 10.1080/01490451.2011.654377

[B11] Diggle S. P. Griffin A. S. Campbell G. S. West S. A. (2007). Cooperation and conflict in quorum-sensing bacterial populations. *Nature* 450 411–414. 10.1038/nature06279 18004383

[B12] Federation and American Public Health Association [APHA] (2005). *Standard Methods for the Examination of Water and Wastewater.* Washington, DC: American Public Health Association (APHA).

[B13] Feltner J. B. Wolter D. J. Pope C. E. Groleau M. C. Smalley N. E. Greenberg E. P. (2016). LasR variant cystic fibrosis isolates reveal an adaptable quorum-sensing hierarchy in *Pseudomonas aeruginosa*. *mBio* 7:e01513-16. 10.1128/mBio.01513-16 27703072 PMC5050340

[B14] Fey A. Eichler S. Flavier S. Christen R. Höfle M. G. Guzmán C. A. (2004). Establishment of a real-time PCR-based approach for accurate quantification of bacterial RNA targets in water, using *Salmonella* as a model organism. *Appl. Environ. Microbiol.* 70 3618–3623. 10.1128/AEM.70.6.3618-3623.2004 15184165 PMC427797

[B15] Foster K. R. Shaulsky G. Strassmann J. E. Queller D. C. Thompson C. R. L. (2004). Pleiotropy as a mechanism to stabilize cooperation. *Nature* 431 693–696. 10.1038/nature02894 15470429

[B16] Gallagher L. A. Manoil C. (2001). *Pseudomonas aeruginosa* PAO1 kills *Caenorhabditis elegans* by cyanide poisoning. *J. Bacteriol.* 183 6207–6214. 10.1128/JB.183.21.6207-6214.2001 11591663 PMC100099

[B17] Garcia-Contreras R. Nunez-Lopez L. Jasso-Chavez R. Kwan B. W. Belmont J. A. Rangel-Vega A. (2015). Quorum sensing enhancement of the stress response promotes resistance to quorum quenching and prevents social cheating. *ISME J.* 9 115–125. 10.1038/ismej.2014.98 24936763 PMC4274435

[B18] Gilbert K. B. Kim T. H. Gupta R. Greenberg E. P. Schuster M. (2009). Global position analysis of the *Pseudomonas aeruginosa* quorum-sensing transcription factor LasR. *Mol. Microbiol.* 73 1072–1085. 10.1111/j.1365-2958.2009.06832.x 19682264 PMC2759405

[B19] Goro K. Yutaro M. Tadashi Y. Koichi H. (2008). Glycine cleavage system: reaction mechanism, physiological significance, and hyperglycinemia. *Proc. Jpn. Acad. Ser. B Phys. Biol. Sci.* 84 246–263. 10.2183/pjab.84.246 PMC3666648 18941301

[B20] Hazan R. Que Y. A. Maura D. Strobel B. Majcherczyk P. A. Hopper L. R. (2016). Auto poisoning of the respiratory chain by a quorum-sensing-regulated molecule favors biofilm formation and antibiotic tolerance. *Curr. Biol.* 26 195–206. 10.1016/j.cub.2015.11.056 26776731 PMC4729643

[B21] Joelsson A. Kan B. Zhu J. (2007). Quorum sensing enhances the stress response in *Vibrio cholerae*. *Appl. Environ. Microbiol.* 73 3742–3746. 10.1128/AEM.02804-06 17434996 PMC1932696

[B22] Kai T. Tateda K. Kimura S. Ishii Y. Ito H. Yoshida H. (2009). A low concentration of azithromycin inhibits the mRNA expression of *N*-acyl homoserine lactone synthesis enzymes, upstream of *lasI* or *rhlI*, in *Pseudomonas aeruginosa*. *Pulm. Pharmacol. Ther.* 22 483–486. 10.1016/j.pupt.2009.04.004 19393329

[B23] Kim M. K. Harwood C. S. (1991). Regulation of benzoate-CoA ligase in *Rhodopseudomonas palustris*. *FEMS Microbiol. Lett.* 83 199–203. 10.1111/j.1574-6968.1991.tb04440.x-i1

[B24] Kim T. S. Ham S. Y. Park B. B. Byun Y. Park H. D. (2017). Lauroyl arginate ethyl blocks the iron signals necessary for *Pseudomonas aeruginosa* biofilm development. *Front. Microbiol.* 8:970. 10.3389/fmicb.2017.00970 28611763 PMC5447684

[B25] Lenney W. Gilchrist F. J. (2011). *Pseudomonas aeruginosa* and cyanide production. *Eur. Respir. J.* 37 482–483. 10.1183/09031936.00122810 21357920

[B26] Miller W. G. Leveau J. H. Lindow S. E. (2000). Improved *gfp* and *inaZ* broad-host-range promoter-probe vectors. *Mol. Plant Microbe Interact.* 13 1243–1250. 10.1094/MPMI.2000.13.11.1243 11059491

[B27] Mitri S. Foster K. R. (2016). Pleiotropy and the low cost of individual traits promote cooperation. *Evolution* 70 488–494. 10.1111/evo.12851 26748821

[B28] Nandi M. Selin C. Brawerman G. Fernando W. G. D. Kievit T. D. (2017). Hydrogen cyanide, which contributes to *Pseudomonas chlororaphis* strain PA23 biocontrol, is upregulated in the presence of glycine. *Biol. Control* 108 47–54. 10.1016/j.biocontrol.2017.02.008

[B29] Neerincx A. H. Mandon J. Van Ingen J. Arslanov D. D. Mouton J. W. Harren F. J. (2015). Real-time monitoring of hydrogen cyanide (HCN) and ammonia (NH3) emitted by *Pseudomonas aeruginosa*. *J. Breath Res.* 9:027102. 10.1088/1752-7155/9/2/027102 25634638

[B30] Park S. J. Kim S. K. So Y. I. Park H. Y. Li X. H. Yeom D. H. (2014). Protease IV, a quorum sensing-dependent protease of *Pseudomonas aeruginosa* modulates insect innate immunity. *Mol. Microbiol.* 94 1298–1314. 10.1111/mmi.12830 25315216

[B31] Passador L. Cook J. M. Gambello M. J. Rust L. Iglewski B. H. (1993). Expression of *Pseudomonas aeruginosa* virulence genes requires cell-to-cell communication. *Science* 260 1127–1130. 10.1126/science.8493556 8493556

[B32] Pessi G. Haas D. (2001). Dual control of hydrogen cyanide biosynthesis by the global activator GacA in *Pseudomonas aeruginosa* PAO1. *FEMS Microbiol. Lett.* 200 73–78. 10.1111/j.1574-6968.2001.tb10695.x 11410352

[B33] Pollitt E. J. G. West S. A. Crusz S. A. Burtonchellew M. N. Diggle S. P. (2014). Cooperation, quorum sensing, and evolution of virulence in *Staphylococcus aureus*. *Infect. Immun.* 82 1045–1051. 10.1128/IAI.01216-13 24343650 PMC3957977

[B34] Sambrook J. Fritsch E. F. Maniatis T. (1989). *Molecular Cloning: A Laboratory Manual.* New York, NY: Cold Spring Harbor Press.

[B35] Sandoz K. M. Mitzimberg S. M. Schuster M. (2007). Social cheating in *Pseudomonas aeruginosa* quorum sensing. *Proc. Natl. Acad. Sci. U.S.A.* 104 15876–15881. 10.1073/pnas.0705653104 17898171 PMC2000394

[B36] Schuster M. Lostroh C. P. Ogi T. Greenberg E. P. (2003). Identification, timing, and signal specificity of *Pseudomonas aeruginosa* quorum-controlled genes: a transcriptome analysis. *J. Bacteriol.* 185 2066–2079. 10.1128/JB.185.7.2066-2079.2003 12644476 PMC151497

[B37] Smalley N. E. An D. Parsek M. R. Chandler J. R. Dandekar A. A. (2015). Quorum sensing protects *Pseudomonas aeruginosa* against cheating by other species in a laboratory coculture model. *J. Bacteriol.* 197 3154–3159. 10.1128/JB.00482-15 26195596 PMC4560282

[B38] Stevens A. M. Schuster M. Rumbaugh K. P. (2012). Working together for the common good: cell-cell communication in bacteria. *J. Bacteriol.* 194 2131–2141. 10.1128/JB.00143-12 22389476 PMC3347067

[B39] Stover C. K. Pham X. Q. Erwin A. L. Mizoguchi S. D. Warrener P. Hickey M. J. (2000). Complete genome sequence of *Pseudomonas aeruginosa* PAO1, an opportunistic pathogen. *Nature* 406 959–964. 10.1038/35023079 10984043

[B40] Toone J. R. Applegarth D. A. Kure S. Coulter-Mackie M. B. Sazegar P. Kojima K. (2002). Novel mutations in the P-protein (glycine decarboxylase) gene in patients with glycine encephalopathy (non-ketotic hyperglycinemia). *Mol. Genet. Metab.* 76 243–249. 10.1016/S1096-7192(02)00041-0 12126939

[B41] van Niel E. W. Claassen P. A. Stams A. J. (2003). Substrate and product inhibition of hydrogen production by the extreme thermophile, *Caldicellulosiruptor saccharolyticus*. *Biotechnol. Bioeng.* 81 255–262. 10.1002/bit.10463 12474247

[B42] Viducic D. Murakami K. Amoh T. Ono T. Miyake Y. (2017). Role of the interplay between quorum sensing regulator VqsR and the *Pseudomonas* quinolone signal in mediating carbapenem tolerance in *Pseudomonas aeruginosa*. *Res. Microbiol.* 168 450–460. 10.1016/j.resmic.2017.02.007 28263907

[B43] Wang M. Z. Schaefer A. L. Dandekar A. A. Greenberg E. P. (2015). Quorum sensing and policing of *Pseudomonas aeruginosa* social cheaters. *Proc. Natl. Acad. Sci. U.S.A.* 112 2187–2191. 10.1073/pnas.1500704112 25646454 PMC4343120

[B44] Waters C. M. Bassler B. L. (2005). Quorum sensing: cell-to-cell communication in bacteria. *Annu. Rev. Cell Dev. Biol.* 21 319–346. 10.1146/annurev.cellbio.21.012704.131001 16212498

[B45] West S. A. Griffin A. S. Gardner A. (2007). Evolutionary explanations for cooperation. *Curr. Biol.* 17 R661–R672. 10.1016/j.cub.2007.06.004 17714660

[B46] Wilder C. N. Diggle S. P. Schuster M. (2011). Cooperation and cheating in *Pseudomonas aeruginosa*: the roles of the *las*, *rhl* and *pqs* quorum-sensing systems. *ISME J.* 5 1332–1343. 10.1038/ismej.2011.13 21368905 PMC3146268

[B47] Xavier J. B. Kim W. Foster K. R. (2011). A molecular mechanism that stabilizes cooperative secretions in *Pseudomonas aeruginosa*. *Mol. Microbiol.* 79 166–179. 10.1111/j.1365-2958.2010.07436.x 21166901 PMC3038674

[B48] Zaara S. Lundgren B. R. Grassa M. T. Wang M. X. Megan G. Moffat J. F. (2016). Erratum for Sarwar et al., GcsR, a TyrR-like enhancer-binding protein, regulates expression of the glycine cleavage system in *Pseudomonas aeruginosa* PAO1. *mSphere* 1:e00200-16. 10.1128/mSphere.00020-16 27471751 PMC4963540

